# Molecular Detection and Genetic Characterization of Avian Leukosis Virus From Field Outbreaks in Bangladesh

**DOI:** 10.1002/vms3.70044

**Published:** 2024-09-23

**Authors:** Md. Golzar Hossain, Riman Pathan, SM Nazmul Hasan, Anandha Mozumder, Moslema Jahan Mou, Marjana Akter, Chandan Sikder, Riyan Al Islam Reshad, Roni Mia, Sukumar Saha, Tofazzal Islam, Sharmin Akter

**Affiliations:** ^1^ Department of Microbiology and Hygiene Bangladesh Agricultural University Mymensingh Bangladesh; ^2^ Institute of Biotechnology and Genetic Engineering Bangabandhu Sheikh Mujibur Rahman Agricultural University Gazipur Bangladesh; ^3^ Department of Physiology Bangladesh Agricultural University Mymensingh Bangladesh

**Keywords:** avian leukosis virus, Bangladesh, genetic characterization, genome sequence, molecular detection

## Abstract

Avian leukosis is a significant viral disease affecting chicken populations globally, including Bangladesh, resulting in high mortality and morbidity rates and causing substantial economic losses in the commercial poultry industry. This study aimed to detect avian leukosis virus (ALV) during recent outbreaks in Bangladesh, utilising a molecular‐based approach. A total of 14 liver samples were collected from the suspected layer flocks in Bangladesh. The diagnosis of ALV infection in chickens was confirmed through necropsy, histopathological examinations, reverse transcription‐polymerase chain reaction (RT‐PCR), and sequence analysis. Gross observations revealed severe liver enlargement with scattered white nodules on the surface in the infected chickens. Histopathological observations showed the infiltration of huge mononuclear inflammatory cells in the periportal area of liver and microvesicular fatty degeneration and necrosis of some hepatocytes. RT‐PCR results identified three samples positive for the *env* gene of ALV. Sequence analysis of the *env* genes demonstrated high homology among the identified strains (97%–98%) and with reference strains (92%–96%) at the nucleotide level. The phylogenetic tree revealed close relatedness of the three identified strains to reference strains from India, USA, and China. Mutational analysis indicated several mutations throughout the envelope glycoprotein of the identified strains. Protein structure analysis showed minor changes in the hydrophobic region of the envelope protein of the identified strains. In conclusion, this study, the first detailed investigation in Bangladesh, contributes to understanding ALV epidemiology, highlights genetic diversity, and emphasises the necessity for further investigations and the implementation of effective control measures in the affected regions.

## Graphical abstract

Histopathological examination of the liver from an avian leukosis virus (ALV)‐infected chicken reveals tissue alterations. Molecular detection confirms the presence of ALV in the liver samples. Evolutionary origin analysis traces the identified ALV strain. Mutational analysis and structural examination of the ALV envelope (Env) protein provide insights into the viral adaptation and function.

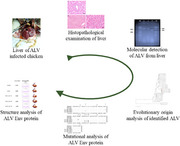

## 1 Introduction

The poultry sector in Bangladesh has been seriously hindered by various infectious and non‐infectious diseases in recent decades, and avian leukosis disease is one of them (Begum, Rahman, and Akter 2016; Hossain et al. 2017; E. Islam et al. 2018; M. S. Islam et al. 2020; Kabir et al. 2021; Mili, Islam, and Al Momen Sabuj 2022). This disease is caused by the avian leukosis virus (ALV), which belongs to the genus *Alpharetrovirus*, subfamily *Orthoretrovirinae*, and is a member of the family *Retroviridae* (Zhang et al. 2020). First reported in the 1920s as a cause of lymphoid tumours in chickens, it has since been recognised as a significant disease in commercial poultry worldwide, including Bangladesh (Sagarika et al. 2017). The virus primarily infects chickens and other birds, including pheasants, partridges, and quails (Plachý, Reinišová, and Kučerová 2017). ALV has been associated with a range of clinical signs and pathological conditions in chickens, such as tumours, immunodeficiency, and decreased egg production (Zhang et al. 2020). The virus targets and infects the bone marrow and lymphoid tissues, leading to the development of tumours and immunosuppression. These tumours can occur in various organs, including the liver, spleen, kidney, and ovary, and they can be either benign or malignant (Dougherty and Di Stefano 1967). One salient feature of this disease is the enlargement of the liver, which occurs due to malignant lymphoid cell infiltration (Mladenov et al. 1967). However, the disease is clinically characterised by inappetence, decreased weight, depression, abnormal feathering, paralysis, and death (Abdellatif and Khalafalla 2005).

ALV is an enveloped virus containing a positive‐sense pseudodiploid single‐stranded RNA (ssRNA) molecule (Appavoo et al. 2015; Buchschacher 2001). The genome of this virus is ∼7.2 kb in length (Freick et al. 2022). The virus genome is non‐segmented and comprises three major coding genes: gag (encoding the internal structural proteins of the virion), pol (encoding RNA‐dependent DNA polymerase), and env (encoding the envelope glycoprotein) (Tang et al. 2022). The virion also codes three enzymes reverse transcriptase (RT), integrase, and protease that are essential for the reverse transcription of viral RNA to DNA copies. These DNA copies integrate into the host genome as a provirus (Venugopal 1999). Based on transmission mode and endogenisation, ALVs are classified into exogenous and endogenous avian retroviruses (Zaib, Hu, and Cui 2022). So far, 11 subgroups (A–K) of ALVs have been identified based on their envelope glycoproteins, virus interference, virus‐serum neutralisation tests, and host range. Among these, A, B, C, D, J, and K are exogenous subgroups naturally found in infected flocks (Tang et al. 2022). Subgroups A and B are common exogenous pathogenic viruses, while subgroups C and D were reported in commercial poultry (Payne et al. 1991). On the other hand, the endogenous subgroup E is either non‐pathogenic or low pathogenic (Ma, Yu, and Chang 2020). This subgroup is transmitted vertically through inherited host genes. However, exogenous subgroups are transmitted both vertically and horizontally (di Stefano and Dougherty 1966).

Various molecular identifications and characterizations of ALVs have been conducted worldwide, including in India, Malaysia, Pakistan, and China (Appavoo, Tomar, and Saxena 2014; Spencer et al. 1984). However, in Bangladesh, only one published report exists on the detection of the viral antigen p27 by ELISA and the isolation of ALV using the egg embryo inoculation method (Begum, Rahman, and Akter 2016). This study was limited to the Dinajpur district in Bangladesh. As of now, there are no reports on the isolation, identification, and molecular characterization of ALV in the country. Therefore, more comprehensive and systematic surveys are needed to gain a better understanding of the scope and impact of the virus on the poultry industry in Bangladesh. In this study, we aimed to identify ALV from a field outbreak by assessing gross and microscopic lesions, employing reverse transcriptase polymerase chain reaction (RT‐PCR), and conducting sequence analyses of the envelope proteins along with predicting their structures.

## 2 Materials and Methods

### 2.1 Study Area and Sample Collection

A total of 14 liver samples from ALV suspected Hy‐Line White layer chicken flocks and one liver from apparently healthy chickens were collected. The ages of the chickens were 20–26 weeks, and the average body weight was 1–1.5 kg. The samples were transported to the Virology laboratory of Microbiology and Hygiene Department, Bangladesh Agricultural University and were then stored at −80°C until used for virus identification.

### 2.2 Sample Processing

Five grams of each tissue (liver) sample were taken, minced using sterile scissors and forceps, and homogenised in a mortar containing sterile sand with a pestle. Phosphate buffered saline (PBS) was added, resulting in a 10% (w/v) suspension. Subsequently, the suspension underwent three cycles of freezing and thawing. The suspension was then centrifuged twice at 3000 rpm for 15 min, and the supernatant was collected. Following this, the supernatant fluid was treated with an antibiotic and antimycotic drugs. Thereafter, the prepared samples were used for ELISA and RNA extraction.

### 2.3 Histopathological Assays

The parts of liver samples were sent to the histopathology laboratory of the Department of Surgery and Obstetrics at Bangladesh Agricultural University for histopathological examination. Subsequently, the liver tissues were dissected, fixed in 10% formalin, embedded in paraffin wax, and subjected to routine staining with haematoxylin and eosin (H&E) following standard procedures for microscopic examination. The stained slides were observed under an OLYMPUS CX41 microscope, and photographs of these slides were captured at the Interdisciplinary Institute for Food Security (IIFS), BAU, Mymensingh.

### 2.4 Enzyme‐Linked Immuno‐Sorbent Assay

A commercially available enzyme‐linked immuno‐sorbent assay (ELISA) kit (Avian Leukosis Virus Antigen Test Kit; BioChek, UK) was utilised in the study. ELISA reagents, including substrate reagent, wash buffer, and samples, were prepared following the manufacturer's recommendations. The test was conducted in accordance with the manufacturer's protocol. The optical density (OD) or absorbance values were determined at 450 nm using an ELISA reader. The mean of the negative control, mean of the positive control, and the sample to positive (S:P) ratio were calculated as per the recommended protocol. The results were interpreted based on the S/P ratio of each sample, with samples having an S/P of 0.3 or greater considered positive and those with an S/P below 0.3 considered negative.

### 2.5 RNA Extraction

The RNA extraction from the prepared inocula was performed using the Monarch Total RNA Miniprep Kit, following the manufacturer's guidelines. The extracted RNA was then stored at −80°C until the reverse transcriptase RT‐PCR was conducted.

### 2.6 One‐Step Reverse Transcriptase Polymerase Chain Reaction

The BioLabs One‐Step RT‐PCR Kit was employed for the reverse transcription PCR process, where reverse transcription and PCR were carried out sequentially in the same tube. All components necessary for both reactions were added during the setup, and the master mix was prepared following the kit's protocol. The reaction components included OneTaq One‐Step Reaction Mix, OneTaq One‐Step Enzyme Mix, gene‐specific primers (ALV‐F: 5′‐GATGAGGCGAGCCCTCTCTTTG‐3′ and ALV‐R: 5′‐TGTGGTGGGAGGTAAAATGGCGT‐3′), nuclease‐free water, and template RNA (Meng et al. 2018). The reverse transcription reaction temperature was set at 48°C for 15 min, followed by activation of the Taq DNA polymerase and inactivation of reverse transcriptase at 94°C for 1 min. Subsequently, the denaturation temperature was set at 94°C for 15 s, the annealing temperature at 62°C for 30 s, and the extension temperature at 68°C for 2 min. The total number of cycles was set at 40. The final extension was performed at 68°C for 5 min.

### 2.7 Sequencing *env* Gene of ALV

Three PCR products that tested positive were sequenced at the “National Institute of Biotechnology” Bangladesh. After sequencing, the obtained data revealed partially amplified *env* gene products with lengths of 212, 201, and 204 bp. The nucleotide sequences derived from these PCR products were aligned with known ALV sequences available in GenBank using NCBI BLAST. Through the BLAST software, homologous nucleotide sequences were downloaded from NCBI GenBank. Finally, the construction of a phylogenetic tree was performed using CLC Sequence Viewer 8.0.

### 2.8 Phylogenetic Tree Construction

The nucleotide sequences obtained from the *env* gene of three identified ALV and 20 isolates from GenBank were subjected to phylogenetic analysis. Our partial *env* gene sequences were substituted in the backbone of the reference strain to contract the phylogenetic tree. The evolutionary history was inferred by using the maximum likelihood method and Tamura 3‐parameter model (Tamura 1992). The tree with the highest log likelihood (−7803.38) is shown. The percentage of trees in which the associated taxa clustered together is shown next to the branches. Initial trees for the heuristic search were obtained automatically by applying Neighbour‐Join and BioNJ algorithms to a matrix of pairwise distances estimated using the Tamura 3 parameter model, and then selecting the topology with superior log likelihood value. The tree is drawn to scale, with branch lengths measured in the number of substitutions per site (next to the branches). This analysis involved 23 nucleotide sequences. All positions with less than 95% site coverage were eliminated; that is, fewer than 5% alignment gaps, missing data, and ambiguous bases were allowed at any position (partial deletion option). There were a total of 1618 positions in the final dataset. Evolutionary analyses were conducted in MEGA11 (Tamura, Stecher, and Kumar 2021). To evaluate trends in the molecular evolution of the ALV‐identified strains, complete *env* gene coding sequences from various ALV reference strains were included in the phylogenetic analysis (Table 1).

**TABLE 1:** Reference ALV strains for the comparison of partial nucleotide sequences of *env* gene of ALV identified strains.

Strain	Year	Location	Gene bank
JS15CRM02	2021	China	MT783289.1
JS13LH14	2021	China	MT624730.1
HB2015032	2018	China	KY581580.1
JS14CZ02	2017	China	KY490696.1
GDFX0601	2015	China	KP686142.1
WD11150b	2018	China	MG812183.1
HLJE2020	2022	China	OK216743.1
DT190904	2020	China	MT319753.1
WB11008e	2012	China	JX570786.1
SD110503R	2014	China	KF738251.1
AF227	2019	USA	MF817820.1
AF229	2019	USA	MF817821.1
JS13LY19	2018	China	MG770235.1
DT190906	2020	China	MT319751.1
DPRE32	2014	India	KM434201.1
WB11098e	2012	China	JX570792.1
RKZ‐2	2022	China	OP856678.1
RKZ‐1	2022	China	OP508143.1
SHPJ21C1	2021	China	MZ836212.1
RAV‐1	1980	USA	MF926337.1

### 2.9 Mutation and Protein Structure Analysis of *env* Gene

For the mutation analysis of the *env* gene at the amino acid level, we initially conducted a BLAST search using our identified sequences. Highly similar sequences were then downloaded and utilised as reference sequences for the mutational analysis. Subsequently, we translated our partial *env* gene sequences by substituting the backbone of the reference strain, which closely resembled our identified strains, and the entire coding regions of the *env* gene of the reference strains into proteins using CLC Sequence Viewer version 8.0. Following this, we aligned our identified sequences with those obtained through the BLAST search, such as MT783289.1, MF817821.1, and KM434201.1. Using the amino acid sequences of the *env* gene from these strains, protein structures were created by the SWISS‐MODEL, which can assist in generating a 3D model based on sequence homology.

## 3 Results

### 3.1 Clinical Signs, Gross, and Microscopic Lesions

**FIGURE 1:**
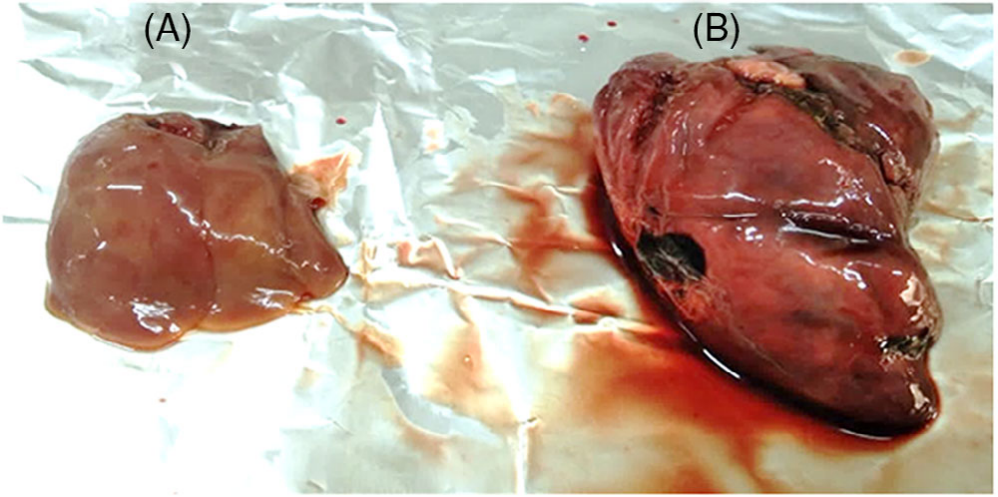
Enlarged liver with characteristic lesion from ALV‐infected chicken. (A) Liver from healthy chicken; (B) multifocal, numerous small, and white nodules scattered on the surface of an enlarged liver of ALV infected chicken.

**FIGURE 2:**
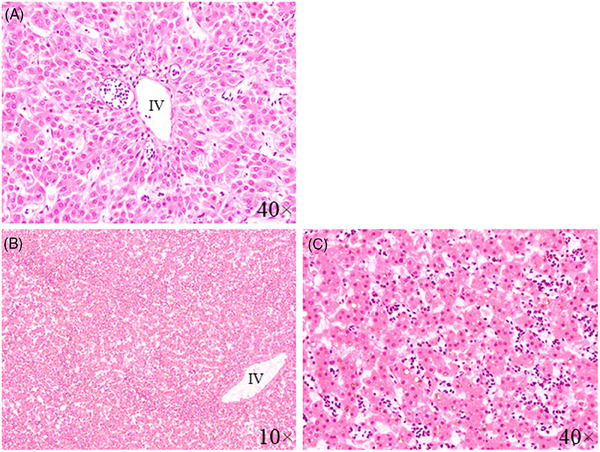
Histopathological lesions of liver of chicken infected with ALV. (A) Liver section from healthy chickens showing a few mononuclear cell infiltrates in the periportal area (H&E stains, 40×). (B) Liver section from infected chicken showing infiltration of huge mononuclear inflammatory cells involving lymphocytes, macrophages, and plasma cells in the periportal area at low power view (H&E stains, 10×). (C) The infected liver section showing microvesicular fatty degeneration and necrosis of some hepatocytes (H&E stains, 40×). IV means interlobular vein.

The diseased chickens exhibited clinical symptoms of ALV infection, including depression, loss of appetite, paralysis, abnormal feathering, weight loss, weakness, diarrhoea, and a decrease in egg production. It was observed that some deceased chickens exhibited numerous small, white nodules scattered on the surface of enlarged livers (Figure 1). H&E staining revealed necrosis and microvesicular fatty degeneration in the of hepatocytes of liver of ALV‐infected chickens. Additionally, the staining showed that infected liver had been infiltrated with huge mononuclear inflammatory cells involving lymphocytes, macrophages, and plasma cells in the periportal area (Figure 2).

### 3.2 Result of ELISA

We conducted ELISA tests from liver inoculum prepared from the infected chickens. The ELISA results indicated that all the samples were negative. However, some wells loaded with liver inoculum showed comparatively higher OD values than others, though not reaching the level of the positive control (data not shown).

### 3.3 Molecular Detection by One‐Step RT‐PCR

**FIGURE 3:**
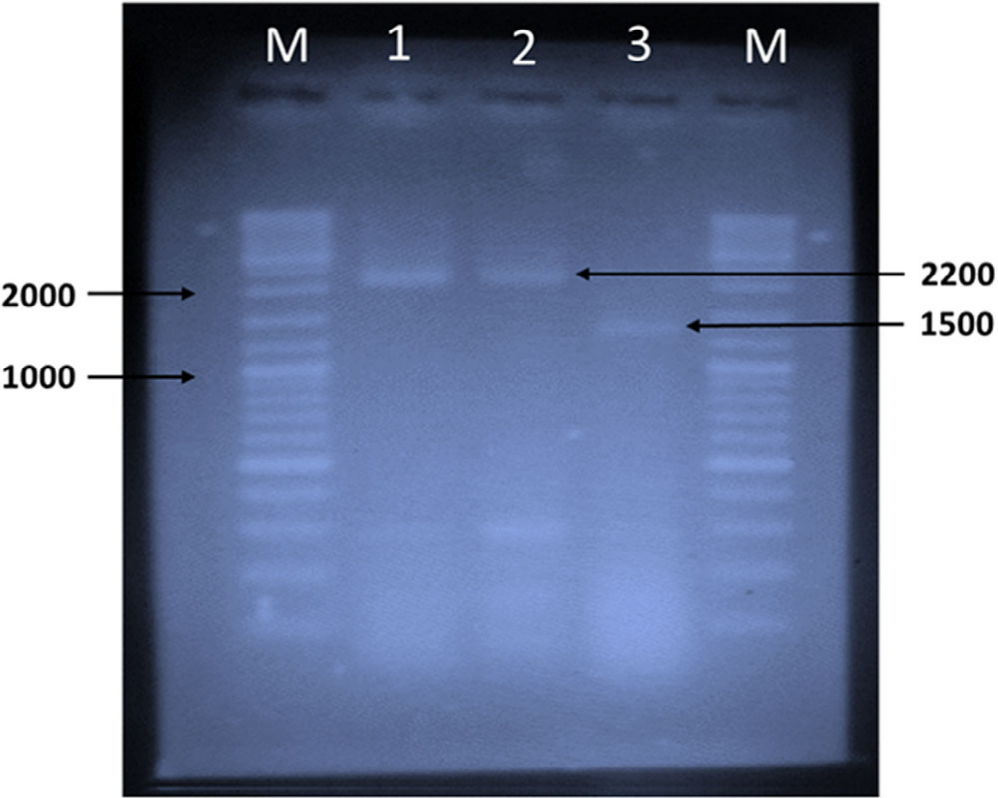
The RT‐PCR products of amplified *env* gene of ALV using *env* gene specific primers. Lane M: DNA molecular weight ladder of 100 bp. Lane 1–2: Positive results, showing 2200‐bp PCR products. Lane 3: Positive results, showing around 1500‐bp PCR product. Lane M: GeneRuler DNA Ladder Mix (#SM0331) was used as DNA marker.

RNA extracted from both the liver inoculum of the infected chickens, as well as from healthy chickens serving as controls, were assayed using one‐step RT‐PCR with *env* gene‐specific primers for ALV. Following gel electrophoresis and visualisation under a UV transilluminator, the results indicated that out of the 14 liver samples, three tested positive. The positive RT‐PCR bands from the samples collected from one farm appeared at the expected ∼2200 bp for the *env* gene of ALV, whereas the samples from another appeared at around 1500 bp (Figure 3).

### 3.4 Sequence Analysis of the *env* Gene

Partial env *gene* sequences were obtained and compared with highly similar sequences from reference strains of the ALV *env* gene. The results revealed nucleotide sequence homology with ALV reference strains ranging from 92% to 96%. When comparing the partial *env* gene sequences of the three identified strains (MGH_ALV‐01, MGH_ALV‐02, and MGH_ALV‐03), it was observed that their genes exhibited significant variation, with nucleotide identities of 97%–98% among themselves. We have submitted these gene sequences to GenBank with the accession numbers OR769215.1 (MGH_ALV‐01), OR769216.1 (MGH_ALV‐02), and OR769217.1 (MGH_ALV‐03).

### 3.5 Phylogenetic Tree Analysis and Evolutionary Relationships

**FIGURE 4:**
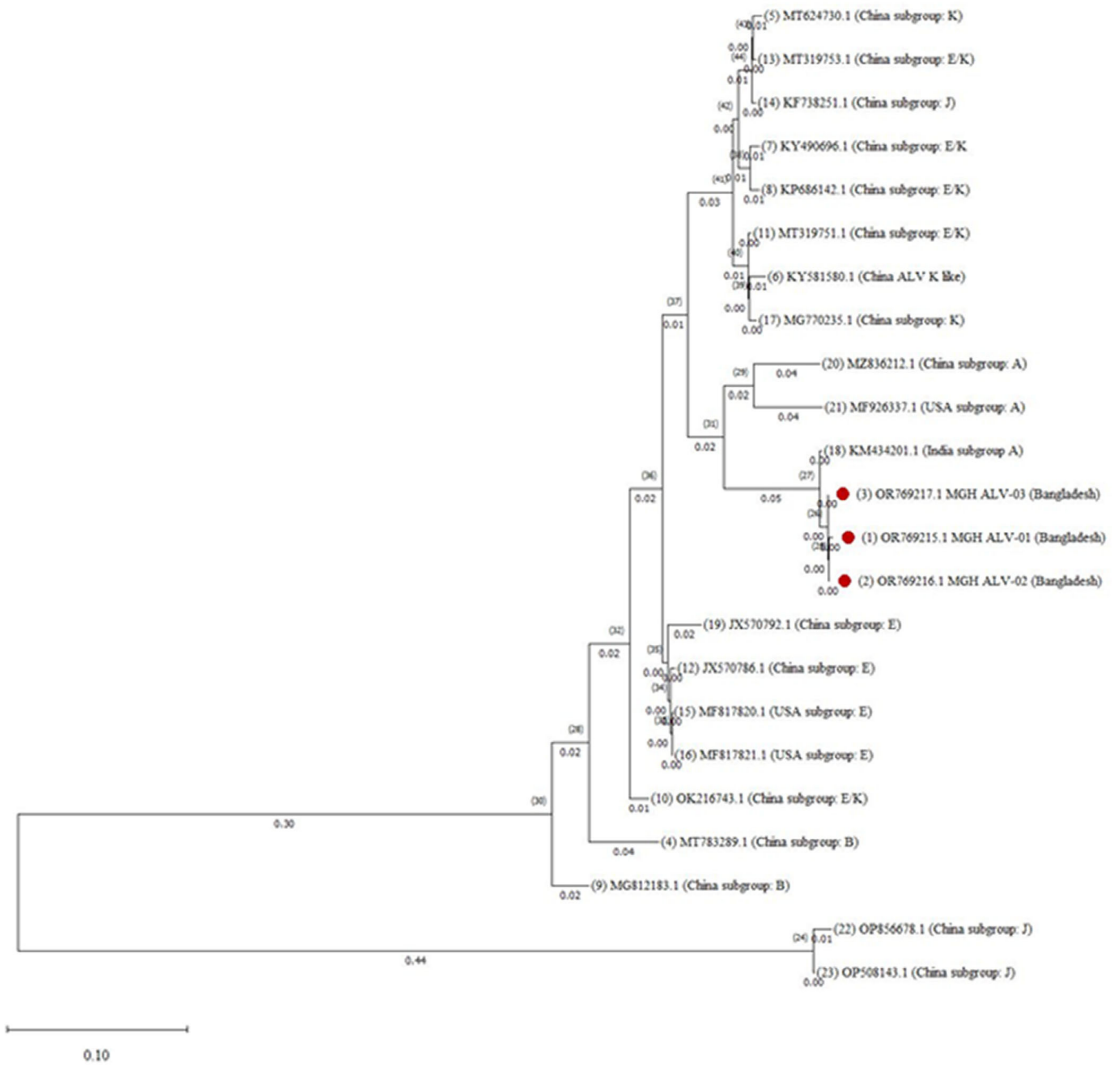
Phylogenetic analysis of the partial *env* gene sequences of ALV‐identified strains in this study. Evolutionary analysis was performed using the maximum likelihood method, Tamura 3‐parameter model, and bootstrapping with a minimum of 1000 replicates, utilising MEGA11 as described in the methods section. The tree is drawn to scale, with branch lengths measured in the number of substitutions per site (next to the branches). The red dot represents this study's identified strains.

Phylogenetic analysis revealed that all three identified ALV strains clustered into an independent group (Figure 4). The tree also indicated that the ALV strains identified in this study were most closely related to chicken isolates of ALV in India and then the United States and China (Subgroup: A).

### 3.6 Mutational Analysis

**FIGURE 5:**
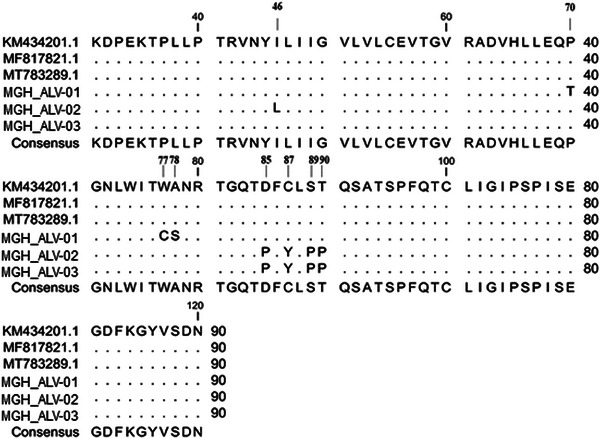
Comparison of the amino acid sequences of *env* gene from ALV‐identified strains and the reference strains of ALV. The dots indicate identical residues, and the letters indicate substitutions.

Mutational analysis revealed several mutations in the surface domain (SU) of the envelope protein of the identified ALV strains. Specifically, MGH_ALV‐01, MGH_ALV‐02, and MGH_ALV‐03 showed three, five, and four amino acid substitutions, respectively, when compared with the sequences MT783289.1, MF817821.1, and KM434201.1, spanning amino acid positions 31–120. MGH_ALV‐02 exhibited the highest number of amino acid substitutions, including the substitution of isoleucine with leucine at position 46. In MGH_ALV‐01, proline, tryptophan, and alanine at amino acid positions 70, 77, and 78 were substituted with threonine, cysteine, and serine, respectively. Additionally, amino acid changes were observed at positions 85, 87, 89, and 90 in both MGH_ALV‐02 and MGH_ALV‐03, where aspartate, cysteine, serine, and threonine were substituted with proline and tyrosine (Figure 5).

### 3.7 Protein Structure Analysis

**FIGURE 6:**
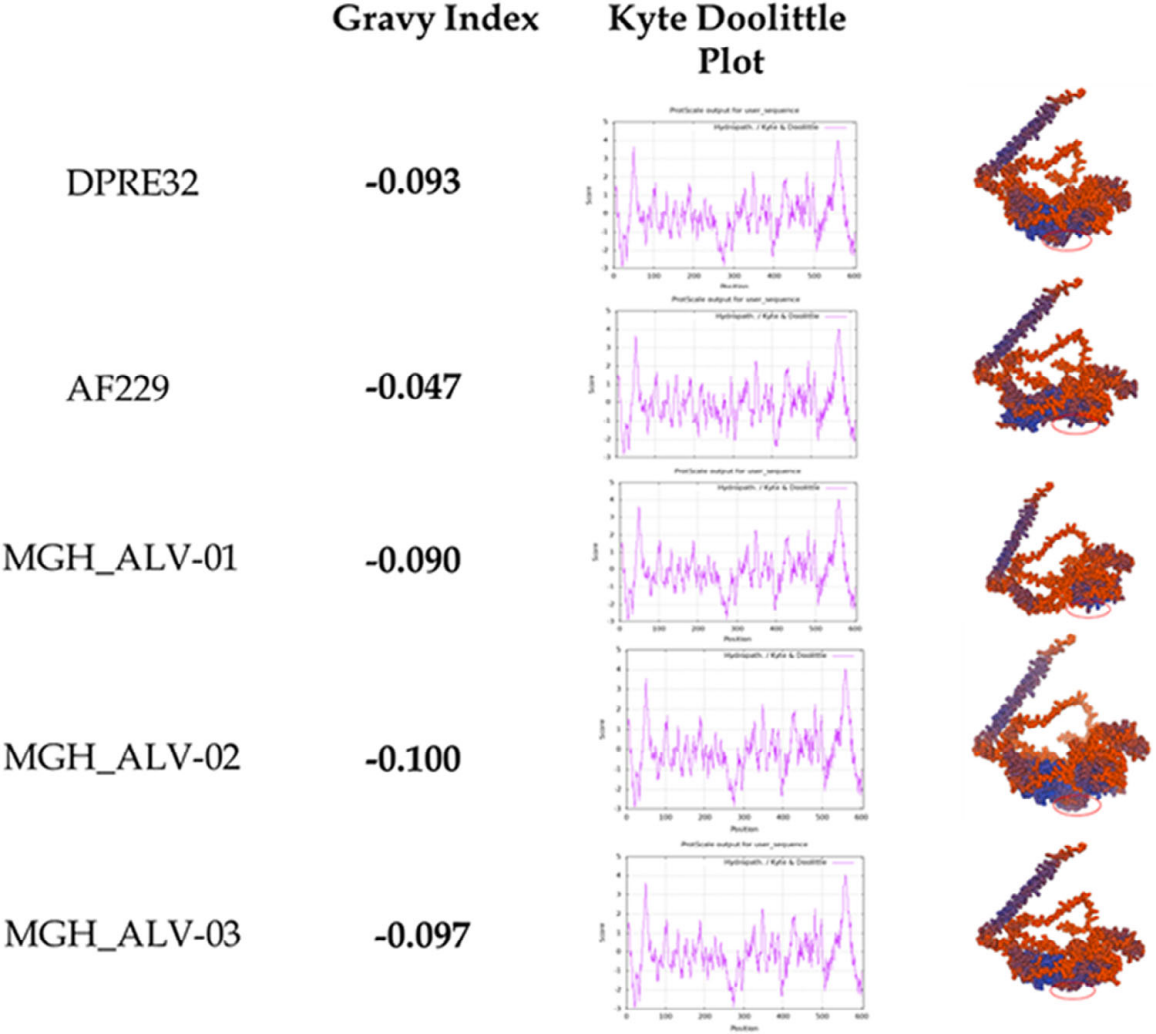
Hydrophobicity analysis of the envelope protein 3D structure of reference and identified strains of ALV. GRAVY Index, Kyte‐Doolittle plots, and surface hydrophobic region (violet) patches are depicted.

Protein structure analysis revealed position‐shifting variations rather than significant differences in the total ratio of hydrophobic surfaces among the identified strains, when compared with reference strains of ALV isolates (Figure 6). GRAVY indexes are reported here for completeness of information; however, they are not suitable for use as evolutionary or functional fingerprints. In fact, variations in GRAVY values among the five envelope protein structures do not correspond to high conservation and fine‐tuning of their surface patches as depicted. Additionally, we noted that the GMQE (Global Model Quality Estimate) value of DPRE32 is 0.07, while other strains have 0.06. The QMEANDisCo global score of AF229 is 0.76 ± 0.06, whereas others have 0.75 ± 0.06. Differences in QMEAN Z‐Scores among the strains are also evident (Table 2).

**TABLE 2:** Comparison protein structure of *env* gene of ALV among reference strains and identified strains.

Parameters	DPRE32	AF229	MGH_ALV‐01	MGH_ALV‐02	MGH_ALV‐03
GMQE	0.07	0.06	0.06	0.06	0.06
QMEANDisCo Global	0.75 ± 0.06	0.76 ± 0.06	0.75 ± 0.06	0.75 ± 0.06	0.75 ± 0.06
QMEAN Z‐Scores	QMEAN	QMEAN	QMEAN	QMEAN	QMEAN
	−0.51	−0.52	−0.51	−0.51	−0.51
	Cβ	Cβ	Cβ	Cβ	Cβ
	3.21	3.13	3.21	3.21	3.21
	All atom	All atom	All atom	All atom	All atom
	3.40	3.44	3.31	3.31	3.31
	Solvation	Solvation	Solvation	Solvation	Solvation
	−0.31	−0.32	−0.33	−0.33	−0.33
	Torsion	Torsion	Torsion	Torsion	Torsion
	−1.26	−1.26	−1.26	−1.26	−1.26

## 4 Discussion

Avian leukosis represents a variety of tumour diseases caused by ALV and sarcoma virus. The presence of ALV in Bangladesh was first detected in 2008 with only one subsequent report on layer chickens infected with ALV (Begum, Rahman, and Akter 2016). The current study focuses on the tentative diagnosis, histopathological assay, ELISA, and RT‐PCR‐based detection of ALV. It includes gene sequencing, phylogenetic analysis, mutational analysis, and predicted structure of envelope proteins of ALV identified from field outbreaks.

ALV can affect various organs in chickens, such as the liver, spinal cord, digestive systems, ovaries, and more (Gavora et al. 1980). Clinical signs observed in affected farms during the outbreaks included inappetence, weight loss, depression, paralysis, decreased egg production, and mortality rates ranging from 1% to 10%. However, the symptoms of avian leukosis depend on the specific organs affected. For instance, inappetence (loss of appetite) may result from damage to the chicken's digestive system (Gavora et al. 1980). Damage to the spinal cord can lead to paralysis in the legs or wings. In laying chickens, the virus can cause tumours in the ovaries, disrupting normal egg production (Hassan and Abdul‐Careem 2020).

ALV can integrate randomly into the host genome, potentially leading to the deregulation of gene expression, particularly affecting regulatory factors like miRNAs (Pajer, Pecenka, and Králová 2006). Abdellatif and Khalafalla (2005) conducted autopsies on sick and deceased broiler chickens, noting hepatomegaly up to three times the normal size—a finding consistent with our results in this study.

In our study, liver of infected chickens was infiltrated with huge mononuclear inflammatory cells involving lymphocytes, macrophages, and plasma cells in the periportal area (Dong, Zhao, and Li 2015; Xu et al. 2022). Such conditions in the liver may arise from the integration of ALV's genetic material into the host cell's genome, leading to the transformation of lymphocytes (a type of white blood cell) into tumour cells (Malhotra et al. 2017). Dong, Zhao, and Li (2015) reported large areas of the liver infiltrated by lymphoid tumour cells and numerous inflammatory cells in affected liver histological sections.

Interestingly, the ELISA report indicated that all samples were negative, while one‐step RT‐PCR revealed three positive samples. Several investigations have addressed similar discrepancies in findings (Brasil et al. 2010; Hendershot, Esayas, and Sutcliffe 2021; Smolejová et al. 2023). An investigation reported positive detection of the p27 antigen of ALV by sELISA (sandwich ELISA) along with PCR, while a commercial ELISA kit showed negative results for all 10 cloacal swabs (Zhou et al. 2019). Xiang, Li, and Liu (2020) found ALV positivity by RT‐PCR in some cell cultures with 0.1 ≤ S/P ≤ 0.2, suggesting that the sensitivity of p27 ELISA was lower than that of RT‐PCR.

The *env* gene of ALV was amplified using gene‐specific primers following Dong et al.’s study, resulting in the identification of three positive samples (Dong, Zhao, and Li 2015). Among them, two RT‐PCR products produced bands at the expected amplicon size of ∼2200 bp, and one appeared at around 1500 bp. The latter may be attributed to the deletion of some nucleotide bases in the ALV *env* gene, as mutations in the template DNA can affect PCR amplification, causing bands to appear at different positions (Zimmermann et al. 2022). Hence, it is plausible that the *env* gene of the identified strain (MGH_ALV‐03) in this study may have some deletions.

The three partially sequenced identified strains of ALV showed close relatedness, with 97%–98% nucleotide identities among themselves and 92%–96% nucleotide identities with reference strains. Phylogenetic analysis indicated that all identified strains in this study fall into a distinct group, suggesting a potential common origin from a single progenitor. This finding aligns with Xu et al.’s report of isolated strains clustering into a special branch (Xu, Mu, and Qian 2021). Notably, these identified strains are most closely related to ALVs from India, USA, China (subtype: A) hinting at possible common ancestors that introduced these strains into Bangladesh.

The envelope protein of ALV is typically cleaved into two functionally distinct domains, SU glycoprotein (N‐terminal subunit) and TM glycoprotein (C‐terminal subunit). The mutation in the envelope protein observed in this study may impact the protein's structure. Protein structure analysis revealed variations in GMQE (Global Model Quality Estimate), QMEANDisCo global score, and QMEAN Z‐Scores values in the identified strains compared to reference strains. QMEAN is a scoring function measuring multiple geometrical aspects of protein structure (Righetto et al. 2014). These changes could alter the protein's overall shape, potentially affecting its ability to interact with host cell receptors and other viral proteins involved in fusion and entry (Yin et al. 2019). Hydrophobicity analysis of the envelope protein structure indicated “position‐shifting” rather than a significant difference in the total ratio of hydrophobic surfaces. However, hydrophobicity plays a crucial role in surface properties, influencing protein interactions (Righetto et al. 2014).

## 5 Conclusions

The clinical signs in ALV infection layer chickens included depression, weight loss, paralysis, and abnormal feathering. Gross and microscopic lesions revealed necrosis in the liver, infiltrated with huge mononuclear inflammatory cells in the periportal area of liver and microvesicular fatty degeneration and necrosis of some hepatocytes. Although ELISA results were negative, one‐step RT‐PCR detected ALV in liver samples, exhibiting variations in sequences. Phylogenetic analysis placed the identified strains in an independent group and have a close relatedness with the ALV subtype A from India, the United States, and China. Mutational analysis showed amino acid substitutions in the envelope protein. Protein structure analysis revealed position‐shifting variations. This study, the first detailed investigation in Bangladesh, contributes to understanding ALV epidemiology and highlights genetic diversity, emphasising the need for ongoing surveillance and control measures.

## Author Contributions

M.G.H. designed study. R.P. and S.M.N.H. conducted the experiments, analysed the data, and prepared an initial draft version of the manuscript. M.G.H. supervised, directed the study, and edited the manuscript. A.M., M.J.M., M.A., C.S., R.A.I.R., R.M., S.S., T.I., and S.A. critically reviewed and revised the manuscript. All the authors edited, revised, and finalised the manuscript.

## Ethics Statement

The authors have nothing to report.

## Conflicts of Interest

The authors declare no conflicts of interest.

### Peer Review

The peer review history for this article is available at https://www.webofscience.com/api/gateway/wos/peer‐review/10.1002/vms3.70044.

## Data Availability

All the data generated in this study are mentioned in this manuscript.
